# Decision-Making Biomarkers Guiding Therapeutic Strategies in Hepatocellular Carcinoma: From Prediction to Personalized Care

**DOI:** 10.3390/cancers17193105

**Published:** 2025-09-24

**Authors:** Dongming Liu, Norihiro Imai

**Affiliations:** 1 Department of Gastroenterology and Hepatology, Nagoya University Graduate School of Medicine, Nagoya 466-8550, Aichi, Japan; 2 Department of Hepatobiliary Cancer, Liver Cancer Research Center, Tianjin Key Laboratory of Digestive Cancer, Tianjin Medical University Cancer Institute & Hospital, Tianjin’s Clinical Research Center for Cancer, National Clinical Research Center for Cancer, Tianjin, China, Tianjin 300060, China

**Keywords:** hepatocellular carcinoma, biomarkers, prediction, immunotherapy, decision, individualized care

## Abstract

Hepatocellular carcinoma (HCC) management has evolved remarkably with the advent of diverse therapeutic options, particularly systemic and surgical treatments. Combination immunotherapy has redefined the treatment paradigm for advanced HCC and contributed to improved patient outcomes. However, this brings forth challenges such as immune-related adverse events that complicate decision-making. Surgical strategies have expanded with the emergence of conversion therapy and borderline resectability, offering curative potential for a broader patient population. However, robust evidence of their long-term efficacy is lacking. Therefore, decision-making biomarkers have gained prominence across treatment modalities. This review explores the current landscape of predictive, prognostic, and treatment-response biomarkers in HCC, from molecular and immune signatures to radiological and biochemical markers, highlighting their role in optimizing therapeutic strategies. By integrating recent advances in basic and translational research with clinical practice, we aim to outline a biomarker-driven framework for individualized care in HCC.

## Simple Summary

Hepatocellular carcinoma treatment has progressed with new systemic and surgical options. Combination immunotherapy improves outcomes for advanced disease but causes serious side effects, complicating treatment choices. Surgical approaches have expanded through conversion therapy and borderline resectability concepts, offering potential cures to more patients, though long-term effectiveness needs confirmation. This changing treatment environment makes biomarkers essential for guiding decisions. This review analyzes current predictive, prognostic, and treatment-response biomarkers, including molecular, immune, imaging, and blood-based types, stressing their role in selecting optimal strategies. By linking recent laboratory and clinical research discoveries, it aims to create a biomarker-driven framework for personalized patient care.

## 1. Introduction

HCC is the most predominant subtype of primary liver cancer, accounting for approximately 75% to 85% of all cases. It originates from the malignant transformation of hepatocytes, the principal parenchymal cells of the liver. HCC remains a significant global health burden, and its epidemiological profile and therapeutic landscape have evolved continuously in recent years. According to GLOBOCAN 2022 data [1], HCC ranks as the sixth most commonly diagnosed cancer globally and the third leading cause of cancer-related deaths. In Asia and Africa, infectious etiologies and aflatoxin exposure dominate as the primary drivers, correlating with the highest global incidence rates [2]. Conversely, in Europe and North America, metabolic factors (metabolic diseases/alcohol consumption) constitute the principal burden, characterized by a lower absolute incidence but a notably upward trend [3]. Prevention strategies must be regionally tailored: high-incidence regions should intensify vaccination and antiviral therapy programs, while low-incidence areas require a shift toward metabolic disease management and alcohol consumption control [4].

Chronic hepatitis B (HBV) and C virus (HCV) infections persist as dominant global risk factors [5]. However, the incidence of metabolic dysfunction-associated steatotic liver disease (MASLD) and metabolic dysfunction-associated steatohepatitis (MASH) leading to HCC has risen significantly in developed countries and some emerging economies, paralleling the epidemics of obesity and diabetes mellitus [6,7]. The incidence of HCC involving alcohol-associated liver disease (ALD) is increasing, as is that of MASLD. ALD represents a range of hepatic disorders comprising steatosis, advanced alcoholic hepatitis, and cirrhosis. These can culminate in liver failure or cancer [8,9]. Antiviral therapies, particularly direct-acting antiviral agents for HCV, tenofovir alafenamide and entecavir for HBV, and universal vaccination are reducing the incidence of virus-associated HCC in some regions [10,11]. Nevertheless, this progress is partially offset by the rapid increase in MASLD/MASH-related HCC [12]. Metabolic syndrome, particularly type 2 diabetes, significantly increases the risk of HCC. Insulin resistance has been identified as the key underlying mechanism [13]. The prognosis of HCC has improved significantly in recent years, an advancement inextricably linked to refined screening strategies and early diagnosis [14]. Target populations, including patients with cirrhosis (irrespective of etiology: HBV, HCV, alcohol consumption, or MASLD) and individuals with advanced hepatic fibrosis, undergo surveillance via abdominal ultrasonography combined with serum tumor biomarkers (alpha-fetoprotein [AFP] and AFP/des-gamma-carboxy prothrombin [DCP]), achieving high sensitivity [15,16]. Early detection improves prognosis primarily through the following mechanisms: (a) maximized access to curative therapies for early-stage HCC; (b) favorable tumor biology characterized by the absence of vascular invasion, low risk of satellite nodules, and reduced microvascular infiltration; (c) preserved hepatic functional reserve, enabling aggressive interventions [17].

Regarding HCC staging systems, the Barcelona Clinic Liver Cancer (BCLC) classification has been conventionally adopted for clinical management [17]. Regional staging systems exhibit variations from the BCLC system, reflecting region-specific therapeutic philosophies and epidemiological profiles [18]. The China Liver Cancer (CNLC) staging system integrates a China-specific national context and extensive clinical experience, comprehensively considering the overall condition of the patient, tumor characteristics, and liver function status. Compared with international staging systems such as the BCLC, the CNLC offers greater granularity and demonstrates enhanced diversity and flexibility in the selection and sequencing of treatment strategies within the multidisciplinary team approach for liver cancer [19,20]. The Japan Society of Hepatology (JSH) released the fifth edition of its Clinical Practice Guidelines for HCC in October 2021. Revised using evidence-based medical methodology, and partially applying the Grading of Recommendations Assessment, Development and Evaluation system, these updated guidelines include new algorithms for systemic therapy, prompted by the expanded range of available drugs, alongside existing surveillance-diagnostic and treatment pathways. This document outlines the revised recommendations and their supporting evidence [21,22]. The key distinctions between the BCLC and CNLC staging systems primarily lie in the surgical threshold of PS scores and the eligibility criteria for liver transplantation. The BCLC staging recommends surgical or interventional treatments only for early to intermediate stage patients with a PS of 0, whereas the CNLC staging extends surgical indications to patients with a PS ≤ 2. Regarding liver transplantation, BCLC restricts its application to stage A and some stage B patients, while CNLC broadens the eligibility to include stage Ia–IIa patients and selected stage IV patients with suboptimal performance status (PS 3–4). In contrast, the differences between the JSH and BCLC staging systems are reflected in the exclusive use of the Child–Pugh classification for performance status assessment. Furthermore, liver transplantation is also considered a treatment option for patients classified as Child–Pugh class B and C under the JSH system. As high-incidence regions for HCC, China and Japan exhibit certain differences in their HCC diagnosis and treatment systems: regarding tumor number stratification, the JSH staging system utilizes thresholds of 1–3 tumors and ≥4 tumors, whereas the CNLC provides a more granular categorization into solitary (one) tumor, two to three tumors, and equal to or greater than four tumors. Concerning tumor size criteria, the JSH system primarily stratifies cases based on a 3 cm threshold, whereas the CNLC employs a 5 cm threshold for solitary tumors but uses a 3 cm threshold for tumors within the two-to-three tumor category. In terms of treatment approaches, the CNLC guidelines incorporate liver transplantation as an option for early-stage HCC, whereas for intermediate-stage HCC (two to three tumors and >3 cm tumor size), the JSH primarily advocates resection and transarterial chemoembolization (TACE) or trans arterial embolization, while CNLC offers additional strategies, such as resection combined with radiofrequency ablation (RFA) or TACE. Finally, for advanced-stage HCC, both systems are largely aligned, with systemic antitumor therapy serving as the primary treatment modality.

Next-generation microwave ablation (MWA) has transcended its historical role as an alternative to RFA, establishing itself as the thermoablative standard for intermediate-sized (3–5 cm) tumors and perivascular HCC. Its synergistic potential with systemic and locoregional therapies underscores an evolving paradigm in precision interventional oncology [23,24,25]. MWA offers the advantage of real-time thermal tumor necrosis but is limited by the heat-sink effect in perivascular lesions [24]. Stereotactic body radiotherapy (SBRT) delivers ablative radiation doses (typically 30–50 Gy in 3–5 fractions) with submillimeter accuracy (≤1 mm precision via robotic collimators), achieving local control rates exceeding 90% for early-stage HCC cases that are unsuitable for resection/ablation and providing durable palliation for locally progressive lesions post-systemic therapy [26,27]. SBRT delivers non-invasive precision, albeit requiring fractionated treatment, yet circumvents vascular heat-sink constraints [27]. TACE remains the cornerstone of treatment for intermediate-stage HCC [28]. Evidence supporting Yttrium-90 (Y-90) radioembolization (selective internal radiation therapy [SIRT] or selective internal radiation embolization) continues to accumulate, demonstrating value in specific cases (e.g., portal vein tumor thrombosis) [29]. Research that compares radio embolization and drug-eluting bead TACE (DEB-TACE) is ongoing [30]. Y-90 radioembolization demonstrates efficacy against portal vein tumor thrombosis while mandating preprocedural lung shunt assessment at the expense of substantial cost. Both conventional TACE and DEB-TACE remain as standards for the management of intermediate-stage HCC, with the latter offering superior sustained drug-eluting properties and durable embolization despite a technically demanding administration [28,29,30].

The evolution of minimally invasive liver resection (MILR), encompassing both laparoscopic and robotic liver resection, has progressively transformed hepatobiliary surgical practice over the past decade. Driven by technological refinements in instrumentation and enhanced surgeon expertise, these approaches are experiencing an exponential adoption for both minor and major hepatectomies [31]. Liver transplantation criteria are being continually refined to expand the eligible population [32]. The oncological expansion beyond the Milan Criteria has been validated in HCC through downstaging protocols [33] (e.g., TACE/Y-90 combined with immunotherapy), enabling successful liver transplantation for patients initially positioned outside the Milan Criteria. The role of neoadjuvant and conversion therapies, particularly immune-based combination regimens, in patients with a high risk of recurrence (resectable or unresectable HCC) is an active area of investigation [34,35]. Technological advancements in MILR have revolutionized major hepatectomies, while concurrently expanding liver transplantation criteria validated by HCC downstaging protocols beyond the Milan Criteria. Emerging neoadjuvant/conversion immunotherapies for high-risk HCC are redefining hepatobiliary oncology paradigms.

The *IMbrave150* clinical trial established the combination of atezolizumab and bevacizumab as the new first-line standard of care for unresectable/advanced HCC, demonstrating significant superiority over sorafenib [36]. The *HIMALAYA* study confirmed that durvalumab combined with a single priming dose of tremelimumab also significantly improves overall survival (OS), establishing another major first-line option [37]. Lenvatinib monotherapy remains an effective alternative [38]. Regorafenib [39], cabozantinib [40], and ramucirumab [41] (for patients with AFP ≥ 400 ng/mL) are established second-line therapies. Agents targeting novel pathways, including those addressing tyrosine kinase inhibitor (TKI) resistance, FGFR4, and TGF-β, are under clinical development [42]. Nivolumab plus ipilimumab is a first-line treatment for unresectable or metastatic HCC in adults, based on comparisons with sorafenib or lenvatinib [43].

### 1.1. The Growing Need for Individualized Treatment Strategies

The rapid development of therapeutic agents with diverse mechanisms of action against HCC necessitates a comprehensive molecular characterization, subgroup biomarker identification, and the establishment of treatment-response databases to enable a rapid selection of optimal regimens for individual patients [44,45]. The Hoshida molecular classification stratifies HCC into three transcriptome-based subtypes with distinct prognostic and therapeutic implications, providing a biological framework for precision oncology beyond anatomical staging systems [46,47,48]. The S1 subtype, characterized by WNT/β-catenin pathway activation and TGF-β signaling upregulation, represents moderately aggressive HCC with a primary resistance to immune checkpoint inhibitors (ICIs). The S2 subtype, defined by MYC/E2F activation, epithelial–mesenchymal transition signatures, and elevated levels of cancer stem cell markers, exhibits the poorest therapeutic outcomes but may derive benefit from AKT/mTOR inhibitors. In contrast, the S3 subtype demonstrates an enrichment of hepatocyte differentiation genes (e.g., HNF4α) and active metabolic pathways, correlating with low invasiveness, enhanced response to sorafenib, and high immunotherapy potential [48]. Consequently, patients with HCC at identical anatomical stages exhibit divergent treatment responses due to underlying molecular heterogeneity [48]. Other molecular classifications of HCC based on multi-omics platforms further substantiate the rationale for individualized therapy. These classifications enable therapeutic stratification transcending conventional tumor node metastasis/BCLC staging systems, underscoring the imperative of precision oncology in HCC management [49].

### 1.2. Role of Biomarkers in Treatment Decision-Making

Biomarkers, serving as objective indicators reflecting tumor biology and disease status, play a critical role in informing treatment decisions for HCC [50,51]. Key biomarkers and clinical scoring systems for HCC diagnosis and prognostic assessment include those enumerated below. AFP, the most widely utilized serum marker, demonstrates 95% diagnostic specificity for HCC at >400 ng/mL, with elevated levels (>1000 ng/mL) doubling the risk of vascular invasion [52,53]. DCP, significantly increased in vitamin K deficiency or HCC, exhibits 65% sensitivity for AFP-negative HCC and serves as an independent predictor of microvascular invasion at >100 mAU/mL [54,55]. The Child–Pugh score, classifying hepatic function into grades A–C (5–15 points) through five parameters (bilirubin, albumin, coagulation, ascites, and encephalopathy), remains a traditional stratification tool for resection and transplant eligibility but is limited by subjective components (e.g., encephalopathy grading). The albumin–bilirubin (ALBI) grade, objectively stratifying liver function into grades 1–3 based on serum markers, overcomes the subjectivity of the Child–Pugh score and more accurately predicts post-TACE liver failure risk [56,57]. The indocyanine green retention rate at 15 min (ICG R15), quantifying functional reserve via dynamic clearance measurement, establishes a critical surgical threshold of ≤10% that permits major hepatectomy (e.g., right trisectionectomy) while an ICG R15 value ≥ 20% restricts resection to subsegmentectomy, guiding operative planning in patients with cirrhosis [58]. The integrated application of these markers (e.g., AFP + DCP enhancing early detection, ALBI + ICG R15 optimizing surgical decisions) is advancing HCC management into a precision oncology paradigm. By uncovering molecular subtypes, predicting therapeutic efficacy, and monitoring drug resistance, they provide an essential basis for selecting, optimizing, and dynamically adjusting treatment regimens, thereby constituting a core element driving the paradigm shift towards precision treatment in HCC [59].

## 2. Biomarkers in Systemic Therapy Decision-Making

### 2.1. Landscape of Systemic Therapies for Advanced HCC Recommendations as First-Line Therapy in Advanced HCC

During the malignant transformation of hepatocytes, multiple signaling pathways are aberrantly activated. Among these, dysregulation of the vascular endothelial growth factor (VEGF), platelet-derived growth factor, fibroblast growth factor (FGF), epidermal growth factor receptor, and hepatocyte growth factor/cellular mesenchymal epithelial transition pathways play critical roles in the development, progression, recurrence, and metastasis of HCC. TKIs have been developed to target these pathways, improving survival in advanced HCC [60]. Programmed Death-1 (PD-1) and its ligand, Programmed Death-Ligand 1 (PD-L1), represent the most established and widely utilized targets for immunotherapy in HCC. Tumor cells evade immune-mediated killing by expressing PD-L1, which binds to PD-1 on T cells, thereby inhibiting T cell activity. PD-1/PD-L1 inhibitors block this interaction, restoring the ability of T cells to attack tumor cells [61]. Cytotoxic T-Lymphocyte-Associated Antigen 4 (CTLA-4) is primarily expressed on the surface of T cells. CTLA-4 binds to B7 molecules on antigen-presenting cells, transducing inhibitory signals that suppress T cell activation during the early stages of T cell priming [62]. Emerging immunotherapeutic targets under investigation include Lymphocyte-Activation Gene 3 (LAG-3), T cell Immunoglobulin and Mucin-domain containing-3 (TIM-3), and T cell Immunoreceptor with Ig and immunoreceptor tyrosine-based inhibition motif domains (TIGIT) [63].

For patients with advanced HCC, first-line treatment options include atezolizumab + bevacizumab (Ate/Bev) or durvalumab + tremelimumab (Dur/Tre) (I, A), sorafenib, lenvatinib, or durvalumab monotherapy (IV, A) [64]. Additionally, on 11 April 2025, the Food and Drug Administration (FDA) approved nivolumab plus ipilimumab for first-line treatment of unresectable or metastatic HCC in adults, based on comparisons with sorafenib or lenvatinib [43]. Ate/Bev therapy significantly improved OS compared with sorafenib: the median OS was 19.2 months versus 13.4 months with sorafenib; median progression-free survival (PFS) was 6.8 months, and the objective response rate (ORR) was 30% [36]. Dur/Tre utilized the single-dose tremelimumab with regular interval durvalumab (STRIDE) regimen, comprising a single high priming dose of tremelimumab combined with durvalumab. The study demonstrated that the STRIDE regimen achieved a median OS of 16.4 months, significantly superior to the 13.8 months observed with sorafenib; the ORR was approximately 20.1%, and the median PFS was 3.8 months. The advantages of this regimen include relatively lower rates of immune-mediated toxicity and the absence of a requirement for anti-angiogenic therapy, making it suitable for patients with a high risk of bleeding [37]. The nivolumab plus ipilimumab regimen, specifically Regimen B (nivolumab 3 mg/kg plus ipilimumab 1 mg/kg Q3W for four doses), achieved a median OS of 23.7 months and an ORR of 36.1%. However, this combination was associated with a higher incidence of immune-related adverse events [43].

### 2.2. Potential Biomarkers in First-Line Treatment of Advanced HCC

ICIs establish a new treatment paradigm for advanced HCC, demonstrating superior efficacy over prior standards with manageable toxicity [62]. ICI-based combination therapies have played a pivotal role in the treatment of advanced HCC, significantly improving patient outcomes and making the prognosis better than before. ICI-based combinations have elevated median OS values to nearly 20 months: a previously unattainable milestone. Future directions include optimizing combination strategies, utilizing predictive biomarkers for patient enrichment, and advancing personalized therapeutic approaches [65]. Current research on biomarkers for first-line treatment of advanced HCC primarily centers on the *Imbrave150* and *HIMALAYA* trials [66,67,68,69,70,71,72,73]. Therefore, we have summarized the key efficacy-associated biomarkers identified in both studies in Table 1.

### 2.3. Predictive Biomarkers of Immunotherapy Response

HCC genomic profiling faces multidimensional challenges. Spatial and temporal heterogeneity impedes comprehensive characterization of tumor clonal architecture through single biopsies [60]. Frequent chromosomal instability and HBV-DNA integration events complicate driver gene identification [74]. Allelic imbalance and copy number variation complexity hinder a precise delineation of key signaling pathways. Host–viral genome interactions in virus-associated HCC remain incompletely elucidated [75]. The absence of standardized multi-omics integration frameworks limits correlative analysis between genomic alterations and phenotypes. Clinical translation is bottlenecked by insufficient prospective validation platforms for establishing molecular classification systems to guide targeted/immunotherapies. Consequently, implementing multimodal biomarker systems is imperative to enhance molecular subtyping precision [76,77,78,79].

### 2.4. PD-L1 Expression, Tumor Mutational Burden, Microsatellite Instability, and Gene Signatures

The immune microenvironment of HCC, driven by tumor heterogeneity, is manifested in two archetypal states: ‘cold tumors’ (immune-deserted) and ‘hot tumors’ (immune-inflamed). Cold tumors are characterized by a CD8^+^ T cell infiltration deficiency, an abundance of immunosuppressive cells (e.g., Tregs, myeloid-derived suppressor cells), and a downregulation of major histocompatibility complex-I gene expression. Their development is primarily associated with Wnt/β-catenin pathway activation, CTNNB1 mutations, or VEGF-mediated aberrant angiogenesis [49]. In contrast, hot tumors exhibit robust CD8^+^ T cell infiltration and functional antigen presentation, typically within pro-inflammatory microenvironments dominated by cytokines like IFN-γ and TNF-α. This dynamic plasticity indicates that HCC immune phenotypes are not fixed entities but exist along a continuous spectrum regulated by molecular heterogeneity and microenvironmental crosstalk [62,80]. Traditional biomarkers for predicting the efficacy of immunotherapy primarily include PD-L1 expression in the tumor microenvironment, encompassing PD-L1 expression on both tumor and immune cell surfaces, as well as tumor mutational burden (TMB) and microsatellite instability (MSI) [81,82]. These three indicators are widely used as predictive biomarkers for immunotherapy efficacy in various cancers. Their application in predicting the efficacy of immunotherapy for HCC has also been extensively explored [83].

Previous clinical studies have investigated whether PD-L1 could serve as a biomarker for predicting immunotherapy response in HCC. The *KEYNOTE-224* trial analyzed the correlation between baseline PD-L1 expression in tumor tissues and treatment response. The results indicated that PD-L1 expression on tumor cells, lymphocytes, and macrophages was associated with response; however, PD-L1 expression solely on tumor cells was not correlated with ORR [84]. This finding was corroborated by the *CheckMate040* study, demonstrating no correlation between baseline tumor PD-L1 expression levels and treatment response [85]. Conversely, analyses from the *CheckMate459* and *GO30140* studies revealed that PD-L1 expression was significantly associated with treatment efficacy and patient prognosis. Higher intratumoral PD-L1 expression levels correlated with increased immunotherapy response rates and improved long-term patient outcomes [86,87]. Therefore, whether PD-L1 can serve as a reliable biomarker for predicting immunotherapy efficacy in HCC remains inconclusive and warrants further exploration.

TMB and MSI serve as biomarkers for predicting immunotherapy efficacy across multiple tumor types [88]. Consequently, numerous previous studies have explored the feasibility of utilizing these two indicators as biomarkers for immunotherapy in HCC. A separate study found a median TMB of 4 mut/Mb across all analyzed specimens, with 95% of samples exhibiting TMB < 10 mut/Mb. Current evidence indicates that a high tumor mutational burden (TMB-H) corresponds to a minority of HCC patients, thereby limiting the clinical applicability of TMB as a predictive biomarker in HCC [89]. Although the National Comprehensive Cancer Network Clinical Practice Guidelines in Oncology for Hepatobiliary Cancers recommend high microsatellite instability (MSI-H)/deficient mismatch repair testing for patients with advanced biliary tract cancers, since they may benefit from pembrolizumab, a genomic sequencing study in advanced HCC cases reported that only 0.2% of patients exhibited MSI-H [90]. Due to this low incidence rate, the utility of MSI status as a predictive biomarker for immunotherapy in HCC is limited, and further research is required to assess its clinical value [91].

With the continuous innovation of novel detection techniques, predictive biomarkers for immunotherapy in HCC are increasingly being identified through genomic, transcriptomic, and proteomic analyses. These include gene mutations, copy number alterations, insertions or deletions, and gene signatures [92,93]. A small-scale study (*n* = 31) reported that a *CTNNB1* mutation was associated with a lower ORR to immunotherapy, shorter mPFS, and shorter mOS values [94]. Another clinical study analyzing 103 patients with HCC receiving first-line combination immunotherapy found no significant correlation between high-frequency mutations (e.g., *TP53*, *TERT*) or TMB and treatment response. However, patients with *MDM4* gene amplification exhibited a markedly reduced response rate to combination immunotherapy [95].

### 2.5. Immune Cell Infiltration and Inflammatory Biomarkers

Immune cell infiltration also plays a crucial role in predicting the response to immunotherapy. Studies indicate that compared to sorafenib, patients with high intratumoral CD8^+^ T cell density demonstrated significantly longer OS values following ‘Ate/Bev’ treatment [68]. Single-cell cytometry by time-of-flight analysis of serial peripheral blood mononuclear cell samples from 10 patients with advanced HCC receiving anti-PD-1 monotherapy revealed distinct longitudinal patterns in major immune subsets differentiating patients attaining a durable clinical benefit from those with a non-durable benefit [96]. Multiple clinical studies have established a strong correlation between inflammatory biomarkers and prognosis in HCC. These biomarkers include the neutrophil-to-lymphocyte ratio (NLR), platelet-to-lymphocyte ratio (PLR), and C-reactive protein (CRP) levels [97,98,99]. Serum amyloid A (SAA), analogous to CRP, is an acute-phase protein. The study demonstrated that hepatocyte-secreted SAA is a pivotal factor mediating resistance to anti-PD-1 immunotherapy in HCC. Consequently, measuring peripheral blood SAA levels holds promise as a predictive biomarker for immunotherapy efficacy in HCC [100].

### 2.6. Biomarkers for Immune-Related Adverse Events (irAEs)

#### Risk Prediction and Early Detection

Although ICIs activate the immune function of the body during the treatment of HCC, they are often accompanied by irAEs [101]. Most irAEs are grade 1 or 2 in severity. However, in rare cases, they can be severe and even life-threatening, such as interstitial pneumonitis and immune-mediated myocarditis [102]. To date, no single biomarker has been proven to reliably predict the occurrence of irAEs [103]. The biomarkers investigated in this regard include those derived from peripheral blood cells, cytokines, tumor genomics, autoantibodies, and fecal microbiology [104]. Among peripheral blood cells, correlations have been observed between the development of irAEs and higher levels of lymphocytes and eosinophils, as well as a lower NLR [105]. The predictive value of cytokines for irAEs primarily manifests through changes in their expression levels following the administration of ICIs [106]. Currently, the cytokines showing the strongest correlations include tumor necrosis factor-alpha (TNF-α), interleukin-6 (IL-6), and interleukin-17 (IL-17). Inhibitors targeting TNF-α and IL-6 are already widely used clinically for the treatment of irAEs. Other candidate cytokine biomarkers under exploration include IL-1, IL-2, IL-8, IL-12, IL-23, granulocyte–macrophage colony-stimulating factor (GM-CSF), interferon-gamma (IFN-γ), CXCL9, CXCL10, CXCL11, and CCL19, among others [107,108,109]. PD-L1 expression levels, TMB, and MSI values are the primary biomarkers clinically used for predicting treatment efficacy [110]. Although primarily predictive of efficacy, these biomarkers also show some predictive potential for the occurrence of irAEs [111]. A pioneering pharmacogenomic study enrolled 307 Asian patients with cancer receiving ICIs, aiming to identify genomic biomarkers associated with irAEs in this population. Initial analysis revealed a novel genetic locus (*LOC105373202 rs5915369)* that was significantly linked to irAEs. Additionally, three independent single-nucleotide polymorphisms (*rs167609*, *rs2341687*, and *DMD rs5928214)* showed a nominal significance [112].

### 2.7. Managing irAEs Through Biomarker-Guided Approaches

Currently, no single biomarker provides precise guidance for predicting irAEs. Biomarker-based management strategies for irAEs aim to achieve preventive intervention. This approach involves identifying high-risk patients and administering pre-emptive therapy to enable a stratified management of the eligible population, thereby avoiding the overuse of immunosuppressants. This strategy helps maintain the intensity of antitumor therapy and ensures adequate exposure to anticancer agents through effective toxicity control [113,114,115,116,117]. Based on this, we summarize the incidence of irAEs (≥grade 3) with the current immune-based combination in the 1st-line of advanced HCC, alongside the types of toxicities (Table 2). Future trends emphasize multi-marker panels over single biomarkers, dynamic monitoring beyond baseline assessment, and the use of integrated data models combining clinical, genomic, and proteomic data with artificial intelligence (AI) [118].

## 3. Surgical Decision-Making and Biomarker Guidance

### 3.1. Conversion Therapy: Expanding Resectability

#### Definition of Conversion Therapy and Its Clinical Relevance

The goal of conversion therapy is to transform patients who were initially unsuitable for surgery or had poor treatment outcomes into candidates amenable to surgical resection, thereby offering the potential for long-term survival to a subset of patients with intermediate and advanced HCC [119]. Resectability is primarily determined by technical and oncological considerations. The former refers to the inability to achieve R0 resection based on imaging assessment, or attain insufficient future liver remnant volume to meet post-operative functional demands [120]. The latter primarily indicates that surgical resection does not offer a better prognosis compared to other non-surgical treatment modalities [120]. Conversion therapy regimens are predominantly derived from advanced-stage treatment protocols, encompassing systemic therapy, locoregional therapy, and combined local–systemic approaches [36,121,122]. These regimens are not contradictory to existing systemic or locoregional therapies; rather, conversion therapy represents an integral component within this continuum. Patients achieving successful conversion gain the opportunity for surgery. Alternatively, for patients without successful conversion, treatment can be promptly switched to second-line strategies, alternative first-line regimens, or combinations with other modalities; participation in clinical trials may also be considered [123].

### 3.2. Outcomes in Patients Undergoing Surgery After Systemic Therapy

The aforementioned *IMbrave150* study established the foundation for targeted-immunotherapy combinations in advanced HCC. Building on this, Kudo et al. [124] found that for patients with advanced HCC unsuitable for TACE, the ‘Ate/Bev’ regimen also achieved a 35% CR rate, with two-thirds of the patients maintaining a recurrence-free status without further treatment during follow-up. Further, multiple retrospective studies [40,125,126] have demonstrated that patients with unresectable HCC and macrovascular invasion have received combination therapy with lenvatinib plus a PD-1 inhibitor. Among those successfully converted and undergoing surgical resection, the 1-year relapse-free survival (RFS) rate was 75% and the 1-year OS rate was 95.8% [127]. Zhang et al. [128] recently published results from a prospective phase II clinical trial demonstrating that following conversion therapy, the 12-month RFS rate was 47.6%, and the median OS after surgery was 31.4 months. The recently published *START-FIT* study [129] is the first to report on the therapeutic potential of sequential TACE or radiotherapy followed by avelumab treatment in advanced HCC cases. Another phase II study involving 36 patients demonstrated that sequential treatment with nivolumab followed by Y-90 achieved an ORR of 41.7%, resulting in a CR rate of 11%. These findings indicate that SIRT holds significant promise for future HCC conversion therapy [130].

### 3.3. The Concept of Borderline Resectable HCC

#### 3.3.1. Imaging Criteria and Multidisciplinary Evaluation

**Figure 1:**
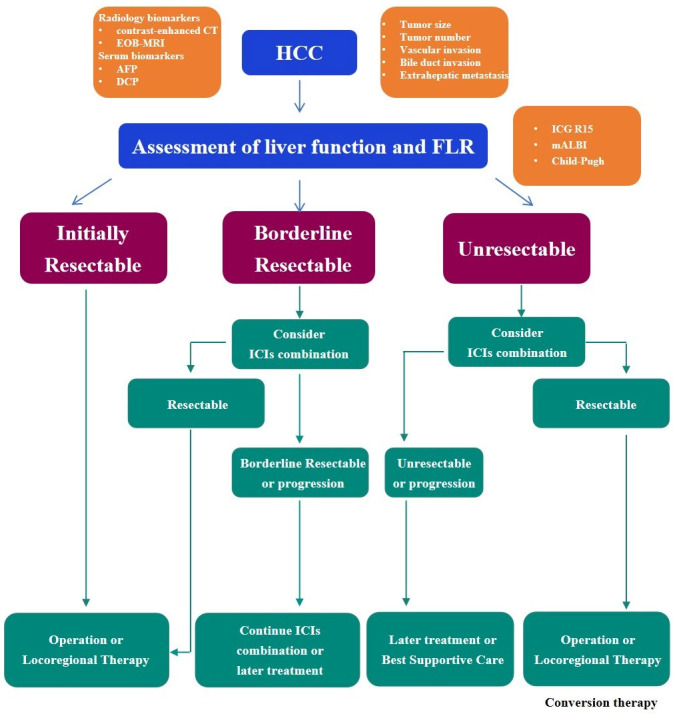
The concept of borderline resectable and conversion therapy.

Amid the growing discourse regarding the therapeutic potential of surgery within multidisciplinary strategies during the course of systemic therapy for advanced HCC [131,132,133], the Japan Liver Cancer Association and Japanese Society of Hepato-Biliary-Pancreatic Surgery released a 2023 Expert Consensus Statement introducing the concept of borderline-resectable (BR) HCC (BR-HCC) [134]. The initial definition for BR incorporated three principal determinants: tumor morphology (size and multiplicity), macroinvasion (involving portal/hepatic veins or biliary structures), and extrahepatic spread [134,135]. This stratification system classifies HCC resectability into three types: resectable (R), borderline resectable1 (BR1), and borderline resectable2 (BR2). BR1 tumors may derive benefit from surgical management within the multidisciplinary strategies employed, whereas BR2 disease denotes an oncological scenario of ambiguous surgical utility necessitating a rigorous multidisciplinary evaluation [136,137]. These criteria aim to establish a unified conceptual framework and terminology to facilitate constructive dialogue between surgeons and oncologists regarding optimal management of advanced HCC cases, a category characterized by marked variations in treatment approaches [138,139]. The concept of borderline resectable and conversion therapy are shown in Figure 1.

#### 3.3.2. Lack of Consensus and the Need for Biomarker-Based Stratification

Although the concept of BR-HCC was first proposed by Japanese researchers, its clinical application has not yet achieved global consensus. The current versions of all major international HCC diagnosis and treatment guidelines have not formally incorporated BR-HCC into their staging systems, leading to significant regional variations in treatment decisions [140]. At the core of this disagreement lies the fact that traditional anatomical criteria are inadequate for accurately assessing the biological aggressiveness of HCC, which is the critical factor determining the benefit from surgery [141]. Further, the assessment of BR-HCC faces several additional shortcomings. First, there is a lack of real-time assessment tools to track the evolution of biological behavior during HCC treatment. Second, a single needle biopsy of a liver tumor often fails to represent the overall characteristics of the tumor due to spatial heterogeneity. Third, tumor immune phenotypes have not been integrated into the assessment [142,143]. Therefore, to address these challenges, the development of a biomarker-based stratification system is urgently needed. However, while serum biomarkers such as AFP, DCP, and circulating tumor DNA (ctDNA) can indicate tumor burden, they possess limited predictive value regarding surgical resectability [144,145]. Looking ahead, integrating anatomical imaging, circulating biomarkers, and AI-driven pathological analysis could facilitate the development of a personalized surgery-indication assessment platform for BR-HCC. Both conversion therapy and borderline resectability hold significant clinical value: by leveraging comprehensive modern therapeutic modalities, they transcend the limitations of traditional surgical indications, enabling more patients at advanced stages to undergo curative-intent surgery, thereby improving their prognosis. This evolution underscores the paradigm shift in oncology management from a purely surgical technique to a multidisciplinary integrated model.

### 3.4. Prognostic Biomarkers for Surgery Candidates

#### Histopathological Features and Molecular Profiles

The efficacy of conversion therapy is a prerequisite for performing liver resection. Therefore, predicting the patient response to conversion therapy and identifying candidates for surgery are particularly crucial [120]. Currently, there is a lack of widely accepted biomarkers for predicting the efficacy of targeted therapy, immunotherapy, locoregional therapy, or combination therapy [118]. However, some reported findings may provide insights. Utilizing patient clinical characteristics, histopathological features, laboratory tests, and imaging examinations to predict the efficacy of systemic antitumor therapy holds some clinical value [127]. Reports indicate that composite scores incorporating serum DCP and metastatic status [146], the ratio of white blood cells to lymphocytes [147], the number of naive CD8^+^ T cells in peripheral blood [148], and radiomics data derived from pre-treatment contrast-enhanced magnetic resonance imaging (MRI) or computerized tomography (CT) scans [148,149] can predict the degree of tumor response to lenvatinib treatment with or without PD-1/PD-L1 inhibitors. Results from one multicenter study showed that pretreatment radiomic features based on dynamic contrast-enhanced MRI correlated with OS and PFS, and could be used to predict the efficacy of conversion therapy in HCC patients [149]. Further, multiple reports suggest that immune cell subsets in peripheral blood may help predict efficacy in patients receiving ‘Ate/Bev’ therapy. Although findings across studies are not entirely consistent, collectively, patients with a high baseline on-treatment NLR tend to exhibit lower tumor response rates, higher incidence of disease progression, and shorter PFS and OS durations [150,151,152,153]. The efficacy of conversion therapy serves as a prerequisite for surgical resection, making the prediction of treatment response and identification of optimal surgical candidates critically important. Although no universally accepted set of predictive biomarkers currently exists, emerging evidence suggests that integrating clinical characteristics, serum biomarkers, peripheral blood indices, and radiomic features can predict tumor response to targeted therapy and immunotherapy. Notably, multicenter studies have confirmed that pretreatment dynamic contrast-enhanced MRI radiomic signatures significantly correlate with OS and PFS time periods, demonstrating a substantial potential for predicting conversion therapy outcomes.

### 3.5. Predicting Recurrence and Long-Term Survival

In the clinical evaluation of conversion therapy efficacy for HCC, a pathological response assessment offers a significant practical advantage by providing relatively accurate early feedback within weeks or months [154]. Preliminary clinical evidence suggests that patients achieving either a major or a complete pathological response exhibit superior postoperative survival outcomes compared with those who do not achieve these responses. Tumor-free survival (TFS) after conversion resection in HCC correlates with the degree of pathological response attained. Patients achieving a pathological response exhibit significantly longer postoperative TFS [155]. Therefore, the hallmark of successful conversion therapy lies not only in achieving resectability but also in assessing the extent of the tumor response, which is more closely associated with postoperative recurrence and long-term survival.

Additionally, several exploratory biomarker findings warrant attention. Studies report that expression of CD274, effector T cell markers, and elevated CD8^+^ T cell density in tumor tissue are associated with a favorable prognosis. Conversely, a high ratio of regulatory T cells to effector T cells and high expression levels of Glypican-3 and AFP are associated with a poor prognosis following treatment [68]. Patients with higher serum levels of soluble CD137 exhibit longer PFS times [156]. Patients with tumors showing a high infiltration-density of M1-polarized macrophages demonstrate higher ORR and longer PFS times [156]. Patients with elevated pre-treatment serum osteopontin (OPN) and AFP levels show higher rates of disease progression upon treatment evaluation and shorter PFS and OS durations [157]. However, it is important to note that these biomarkers still lack sufficient clinical validation. A summarized version of these results is presented in Table 3.

## 4. Biomarkers Across the Disease Continuum

### 4.1. Predicting Disease Progression and Relapse

#### ctDNA, AFP Dynamics, and Methylation Markers

Cell-free DNA (cfDNA) refers to fragments of nucleic acids released into the bloodstream, originating from apoptotic, necrotic, and normal eukaryotic cells. ctDNA constitutes a subset of the total cfDNA pool [76]. It is precisely these cancer-specific genetic and epigenetic changes within ctDNA that have captured the interest of researchers exploring novel biomarkers for various cancers, including HCC [158,159,160]. Previous studies have indicated that ctDNA molecules in the peripheral blood of patients with HCC exhibit a greater variability in size and are shorter [77,161]. A detailed analysis of plasma DNA size distribution suggests that short DNA molecules may preferentially carry tumor-associated genetic mutations. Qu et al. [162] developed a liquid biopsy method named HCC screen, based on the detection of ctDNA somatic mutations combined with protein biomarkers. This assay effectively identified early-stage HCC within a high-risk subgroup from a large community-based cohort.

AFP demonstrates high specificity but low sensitivity for the diagnosis of early-stage HCC. Research has shown that in patients with cirrhosis, using an AFP threshold of 20 ng/mL yields a sensitivity of 41–65% and a specificity of 80–94% for diagnosing HCC across various stages [52]. At a lower threshold (10.9 ng/mL), the sensitivity for detecting early-stage HCC increases to 66%, with a specificity of 82% [53]. To enhance the sensitivity of AFP for HCC diagnosis, it is necessary to combine AFP testing with other biomarkers. Currently, the GALAD model, which incorporates three biomarkers (AFP, AFP-L3, and DCP), and the ASAP model, which combines AFP, DCP, and several clinical parameters, have been shown to significantly improve the diagnostic performance for early-stage HCC [163,164]. Therefore, it is particularly important to utilize dynamic AFP levels as a reference for predicting prognosis in HCC. Yang et al. [144] enrolled 536 patients with advanced HCC treated with bevacizumab combined with ICIs. By grouping patients based on pre-treatment AFP levels, they demonstrated for the first time that AFP trajectory classification outperformed conventional biomarkers in predictive efficacy. The data indicate that a rapid decrease in AFP levels (>90%) during the initial phase of treatment (within 4 months) was the strongest predictor of survival benefit. Crucially, even if subsequent biochemical recurrence occurred, the prognosis for these patients remained significantly superior compared to other trajectory types.

Traditionally, the analysis of cfDNA has focused on measuring its total concentration, integrity, and copy number alterations [76]. In contrast, newer methodologies probe methylation patterns, specifically targeting CpG islands of tumor suppressor genes, or identifying mutational signatures that reflect the current “tumor footprint” of the patient [165]. DNA methylation, a well-characterized epigenetic modification, serves as a key epigenetic regulator of gene expression [166]. It is intricately linked to DNA regulation and cancer pathogenesis. Critically, aberrant DNA methylation can occur during the early stages of tumor development, offering potential value for the early diagnosis of HCC [167]. The HelioLiver test, which integrates the methylation profile of cfDNA across 28 genes (covering 77 CpG sites) with patient age, sex, and three serological biomarkers (AFP, AFP-L3, and DCP), achieved an overall sensitivity of 85.2% and specificity of 91.2%. This study cohort included 122 patients with HCC and 125 control individuals with benign liver disease. The test demonstrated an early-stage cancer detection sensitivity of 76%. Critically, its sensitivity for detecting both overall and early-stage HCC surpassed that of AFP, AFP-L3, DCP, and the GALAD model [145]. The Oncoguard^®^ Liver test diagnoses early HCC via a multitarget serum assay measuring a panel of methylation markers and serum biomarkers. In a 2021 study, this assay utilized four methylation markers along with AFP and AFP-L3. It reported an overall sensitivity of 80%, an early stage detection sensitivity of 71%, and a specificity of 90%, demonstrating superior sensitivity compared to AFP, AFP-L3, DCP, and GALAD [168]. Additionally, in a separate cohort of 156 patients with HCC and 245 control individuals, the test achieved an overall sensitivity of 88%, an early-stage detection sensitivity of 82%, and a specificity of 87% [169]. ctDNA, AFP, and methylation markers are pivotal tools for predicting HCC progression. Each biomarker offers distinct advantages and differs in its predictive value, while also presenting specific application challenges. The integration of these three markers holds significant promise for enabling individualized recurrence risk assessment, guiding precision interventions, and ultimately improving survival outcomes.

### 4.2. Early Detection of Recurrence Through Liquid Biopsy

Beyond the classic liquid biopsy modalities mentioned above, such as cfDNA and ctDNA, noncoding RNAs (ncRNAs), extracellular vesicles (EV), and exosomal circular RNAs also hold significant value for the early diagnosis of HCC [76,78,79]. The primary constituents of ncRNAs are microRNAs (miRNA) and long ncRNAs (lncRNAs). These are RNA molecules that are not translated into proteins and serve as crucial regulatory elements involved in chromatin modification, DNA transcription, and chromosome looping. Studies have shown significantly elevated serum levels of miRNA-122 in patients with early-stage HCC compared with healthy controls [170]. Research by Ding et al. [171] indicated that the detection of miR-122-5p in circulation suggests the early occurrence of HCC. Further, Li et al. [172] demonstrated that combining miRNA profiling with AFP levels significantly enhances the efficacy of early HCC detection by improving both sensitivity and specificity [173]. Serum levels of *lncRNA SCARNA10* were significantly higher in patients with HCC compared with both benign liver disease groups and healthy controls. Moreover, *lncRNA SCARNA10* levels exhibited a positive correlation with the degree of tumor malignancy [174].

EVs are membrane-enclosed structures released by cells and detectable in plasma. They carry a diverse biochemical cargo, including genetic material, and have been investigated as biomarkers for the early detection of HCC [75,175,176]. In one study comparing plasma from 36 patients with early-stage HCC and 26 control individuals with cirrhosis, an EV-based assay demonstrated a sensitivity of 94.4% and a specificity of 88.5% [177]. Further, Sun et al. [178] developed an early HCC screening model based on the detection of EV surface proteins. This model exhibited exceptionally high accuracy in discriminating early-stage HCC from cirrhosis. Lin et al. [179] found that *circ_0072088* was upregulated in HCC tissues and cells compared with adjacent non-tumor tissues and healthy hepatocytes, and exhibited high expression in plasma exosomes derived from patients with HCC. Kaplan–Meier survival curves and Cox proportional hazards model analysis revealed that patients with HCC with elevated exosomal *circ_0072088* expression had a poorer prognosis. Liquid biopsy enables early detection of HCC recurrence by identifying molecular residual disease months before radiographic progression. ctDNA variants and tumor-specific methylation signatures offer a high specificity for predicting relapse. Integration of these biomarkers may guide preemptive therapy, potentially improving survival.

### 4.3. Integration of Imaging Biomarkers

#### Radiomics and Functional Imaging

In HCC diagnosis and staging, multiphasic contrast-enhanced computed tomography (CT) serves as the first-line modality for detecting > 1 cm lesions, characterizing the pathognomonic “wash-in/wash-out” enhancement pattern, evaluating vascular invasion, and guiding surgical planning. Dynamic enhancement patterns further differentiate malignancies: arterial hyperenhancement with washout supports an HCC diagnosis, while peripheral nodular enhancement suggests the presence of hemangioma, and homogeneous enhancement with central scar indicates focal nodular hyperplasia [180]. Gadolinium-ethoxybenzyl-diethylenetriamine pentaacetic acid enhanced MRI [181], the gold standard for detecting solitary HCC lesions ≤ 2 cm in size, leverages hepatobiliary-phase hypointensity to confirm HCC and distinguishes early HCC from dysplastic nodules. Additionally, it assesses hepatic functional reserve via parenchymal enhancement. For systemic evaluation, ^18^F-fludeoxyglucose-based positron emission tomography-CT [182] excels in detecting extrahepatic metastases, identifying aggressive HCC (maximum standard unit value > 3.5), and monitoring metabolic treatment response. Collectively, these complementary techniques enable comprehensive HCC management from early detection to metastatic surveillance.

The concept of radiomics, proposed by Lambin et al. in 2012 [183,184], involves the high-throughput extraction of numerous features from radiological images. Utilizing statistical and AI algorithms, radiomics identifies the most valuable features to construct predictive models for tumor diagnosis, treatment, and prognosis assessment. Radiomics has progressively become an integral component of intelligent diagnostics, offering novel opportunities for the diagnosis and treatment of HCC.

HCC radiomics research primarily utilizes ultrasound (US), CT, and MRI. Although US demonstrates a diagnostic performance comparable to CT for small HCCs, its application is limited. CT and MRI are more commonly employed for HCC diagnosis and recurrence prediction, exhibiting similar predictive performances [185]. Among traditional HCC screening tools, despite its widespread use, US has a sensitivity of only 47% for early-stage HCC, with significant limitations in accurately identifying small lesions [186]. Although CT and MRI offer higher resolution, their ability to identify early-stage lesions remains constrained, particularly in precisely assessing lesion heterogeneity [187].

Radiomics-based diagnostic models perform comparably to experienced radiologists, and may even outperform them in certain clinical scenarios [188]. Bharti et al. [189] developed a neural network model based on US images to differentiate the four stages of HCC development, achieving a classification accuracy of 96.6%. Radiomics technology enables the non-invasive preoperative assessment of HCC pathological characteristics and assists in optimizing treatment strategies. Radiomics models exhibit exceptional capability in predicting HCC histopathological grade [190,191]. Wei et al. [192] developed a deep-learning radiomics model by extracting features from both the tumor and peritumoral regions of contrast-enhanced CT images of patients with HCC. Their results demonstrated that peritumoral features hold significant value in reflecting the tumor microenvironment and are closely associated with histological grade.

Radiomics also plays a pivotal role in treatment decision-making for unresectable HCC, showing promise in the context of TACE. Li et al. [193] demonstrated that combining tumor growth patterns on contrast-enhanced MRI, radiomic features from both the tumor and peritumoral regions, and the ALBI score enables non-invasive and personalized prediction of the TACE response in patients with HCC. Radiomics holds significant value in evaluating systemic therapy efficacy, aiding in the identification of patient subgroups most likely to benefit and improving treatment targeting. Xie et al. [194] established a self-supervised contrastive deep learning model capable of accurately predicting PD-1 and PD-L1 expression in HCC cases. This non-invasive approach can guide the precise application of ICIs in HCC. Radiomics demonstrates substantial value in predicting HCC survival outcomes. MRI-based radiomics studies indicate that features derived from the tumor and surrounding regions can effectively predict PFS in patients with early-stage HCC following RFA [195]. Further, a study integrating contrast-enhanced MRI radiomics with clinicopathological factors [196] developed a model predicting 3-year OS post-hepatectomy for HCC cases.

### 4.4. Linking Imaging Phenotypes to Molecular Profiles

Currently, the frontier of radiomics also encompasses its integrated application with other -omics disciplines. Pathomics [197], as an emerging tool, enables comprehensive feature extraction and improves tumor prognosis assessment. Further, its integration with radiomics can further enhance model performance. Feng et al. [198] developed and validated a radiopathomics model combining MRI radiomic features with whole-slide pathological images to predict OS in HCC cases. The resulting nomogram demonstrated excellent predictive accuracy and clinical utility, achieving C-indices of 0.840 and 0.875 in the training and validation cohorts, respectively.

Genomics elucidates the pathogenesis of HCC at the molecular and genetic levels through gene mapping, localization, and functional analysis. A radiogenomics approach links imaging features to key genes and pathways, providing biological annotation for models and enhancing their interpretability. Wang et al. [199] developed a CT-based radiomics model that identified seven OS-related genes associated with tumor microenvironment heterogeneity and TACE response, successfully predicting OS duration and treatment response.

Additionally, a study on a CT-based radiomics nomogram [200] showed that the model could not only predict proliferative HCC but also forecast RFS or PFS in stratified patients following surgery or intra-arterial chemoembolization. Importantly, this model exhibited significant correlations with HCC carbon metabolic pathways, immune cell infiltration, and tumor heterogeneity. Such multimodal research integrating radiomics, pathomics, genomics, and other disciplines holds significant importance for unraveling HCC morphobiology and enhancing prognostic assessment capabilities [201,202].

### 4.5. Blood-Based Biochemical Markers

#### ALBI Grade, DCP, NLR, and Composite Scoring Systems

ALBI grade and the platelet-albumin-bilirubin (PALBI) grade have been reported as effective objective indicators for assessing liver functional reserve. A study by Hiraoka et al. [56] demonstrated that the ALBI grade provides a more precise and comprehensive assessment of liver function in HCC cases compared with the Child–Pugh score. Consequently, it is considered the optimal alternative to the Child–Pugh score. Liu et al. [203] discovered that the PALBI grade outperforms the ALBI grade, model for end-stage liver disease, and the Child–Pugh score in assessing liver function. Most recently, Ho et al. [57] established the ALBI-HOME prognostic risk model based on the ALBI grade for patients beyond the Milan criteria. It holds promise for guiding more effective treatment strategies for high-risk patients and may have positive implications for the evaluation of conversion therapy.

DCP, also known as PIVKA-II, is detectable in the serum of individuals with vitamin K deficiency or patients with HCC. Elevated serum DCP levels (≥7.5 ng/mL) are associated with a five-fold increased risk of developing HCC. Based on this, DCP has been approved by the FDA for risk assessment [54]. A meta-analysis incorporating 83 studies demonstrated that DCP has a sensitivity of 0.71 and a specificity of 0.91 for diagnosing HCC [55].

It is currently recognized that the development and progression of HCC are closely associated with inflammatory responses. Both the NLR and PLR, as inflammation-related markers, have been reported to correlate with poor prognosis in HCC. Through data analysis, Zeng et al. [204] found that a higher preoperative NLR level was positively correlated with the risk of microvascular invasion, which is considered a significant indicator of intrahepatic tumor metastasis [205]. Rungsakulkij et al. [206], analyzing data related to PLR, also reported corresponding results: a larger preoperative tumor size and an elevated PLR level were independent predictors of microvascular invasion following hepatectomy. A study by Zheng et al. [207] indicated that pre-treatment values of both NLR and PLR were independent risk factors for OS and RFS in HCC cases, regardless of whether they undergo curative or palliative therapy. Using an internal validation cohort of 360 patients with cirrhosis and an external validation cohort of 2700 patients with cirrhosis, Wang et al. [208] demonstrated that the Doylestown model significantly improves the detection rate of HCC compared with using AFP alone.

## 5. Conclusions

### 5.1. Summary of Current Evidence

The utility of biomarkers in liver cancer primarily advances the following domains: (a) Disease Detection and Prognostic Assessment: the combined detection of serum AFP-L3 fraction and DCP can effectively identify patients at high risk of recurrence post-curative resection, thereby guiding intensified surveillance strategies. (b) Targeted Therapy Guidance: VEGFA gene amplification status correlates with sensitivity to anti-angiogenic agents, while an FGF19 overexpression indicates a potential resistance to specific TKIs. (c) Immunotherapy Stratification: A TMB coupled with tumor microenvironment immune cell infiltration characteristics approach enables a more precise identification of beneficiaries for ICIs than PD-L1 expression alone. (d) Dynamic Therapeutic Monitoring: molecular residual disease analysis via ctDNA allows an earlier detection of treatment response compared with conventional imaging, driving real-time adjustment of clinical intervention strategies. The current evidence framework has begun to establish a “prediction–intervention–reevaluation” closed-loop decision-making system. However, further optimization of clinical translation pathways through multimodal biomarker integration remains imperative.

### 5.2. Challenges and Future Opportunities

The field continues to face significant challenges. Biomarker heterogeneity: dynamic evolution of the tumor microenvironment results in pronounced spatiotemporal heterogeneity of biomarkers, limiting the predictive efficacy of single-marker approaches. Clinical translation bottleneck: emerging biomarkers (e.g., ctDNA methylation, multi-omics signatures) lack standardized detection protocols and large-scale prospective validation. Inadequate decision system integration: the association between established biomarkers (e.g., PD-L1, TMB) and treatment response suffers from threshold ambiguity, and a dynamic monitoring-driven closed-loop decision-making system remains unrealized.

Future directions for biomarkers guiding personalized HCC therapy should focus on three dimensions. Multimodal integration: Constructing “molecular-imaging” dual-track predictive models using spatial transcriptomics and radiomics to enhance early treatment response assessment accuracy. Dynamic monitoring innovation: Liquid biopsy-driven real-time tracking of ctDNA mutational profiles to guide sequential regimen modifications in targeted therapy and immunotherapy. Intelligent decision systems: AI-based integrated analysis platforms that synthesize genomic, pathological, and clinical data to generate personalized therapeutic roadmaps, ultimately enabling a paradigm shift from “static classification” to “dynamic intervention”. However, in this process, investigators should be mindful of the impact of overfitting risk on machine learning and biomarker research, employ multiple strategies such as cross-validation and internal validation, and interpret the findings with caution. Additionally, future research should focus on both exploring novel biomarkers and conducting clinical trials to validate established ones.

### 5.3. Toward a Biomarker-Driven Clinical Decision-Making Model

Current research is shifting HCC treatment decisions from experience-based to biomarker-driven paradigms:

- Multimodal data integration: This combines genomic variants, immune microenvironment features, and radiomics to construct adaptive risk stratification models.
- Closed-loop decision mechanism: This establishes a “treatment-response assessment-therapy adjustment” feedback cycle.
- Intelligent analytics engine: This leverages AI algorithms to analyze multi-omics data streams, generating personalized therapeutic roadmaps that transcend population-based guidelines. This model transforms static biomarkers into dynamic decision variables, achieving an organic integration of predictive monitoring and pre-adaptive therapy.

## Figures and Tables

**Table 1:** Potential biomarkers in first-line treatment of advanced HCC.

Category/Biomarker	Key Findings
AFP	
Baseline AFP levels	In the *IMbrave150* study, baseline AFP levels were a key stratification factor. Patients with AFP < 400 ng/mL may derive more significant benefit from Ate/Bev therapy compared with those with AFP ≥ 400 ng/mL.
Post-treatment AFP changes	Exploratory analyses of the *IMbrave150* studies revealed that a ≥75% reduction or ≤10% elevation in AFP levels at week 6 post-treatment was significantly associated with improved OS and PFS.
DCP	
Post-treatment DCP changes	In the *HIMALAYA* study, the results demonstrated that patients exhibiting a DCP reduction of >40% at week 4 achieved treatment response in approximately 72% of cases.
Tumor-related genes and protein expression	
CD274 and TEFF	In the *IMbrave150* study, high pre-existing expression of CD274 and TEFF was associated with greater benefit from Ate/Bev therapy. Patients with complete CR/PR had higher expression of ABRS, CD274, and TEFF than those with stable disease/progressive disease (SD/PD).
CD8^+^ T cells	In the *IMbrave150* study, the concentration of CD8^+^ T cells in tumor tissue correlated with PFS and OS benefits from Ate/Bev therapy. In the *HIMALAYA* study, elevated levels of CD8^+^ T cells were associated with superior response rates to the STRIDE regimen.
TREG/TEFF ratio	A low TREG/TEFF ratio was associated with more significant improvements in PFS and OS after Ate/Bev therapy.
Wnt/β-catenin Signaling Pathway	Patients with inactivation of the Wnt/β-catenin signaling pathway showed enhanced response rates to the STRIDE regimen.

Note: AFP, alpha-fetoprotein; OS, overall survival; PFS, progression-free survival; DCP, des-gamma-carboxy prothrombin; CR, complete response; PR, partial response; SD, stable disease; PD, progressive disease.

**Table 2:** The irAEs in the 1st-line therapy (immune-based) of advanced HCC.

1st-Line Therapy	Incidence ≥ G3 irAEs	Common irAEs
*IMbrave150*	36.0%	Hypertension AST increased ALT increased
*HIMALAYA*	25.8%	AST increased Lipase increased Amylase increased
*CHECKMATE-9DW*	41.0%	AST increased ALT increased Lipase increased

Note: AST: aspartate aminotransferase; ALT: alanine aminotransferase.

**Table 3:** Surgical decision-making biomarkers.

Predictive Scenarios	Biomarker Category	Biomarker
Prognostic Biomarkers for Surgical Candidates	Tumor biomarkers	AFP
		DCP
		ctDNA
	Imaging Response Markers	RECIST or mRECIST
		contrast-enhanced MRI
	Liver Functional Reserve Markers	ICG-R15
		ALBI
Postoperative recurrence and long-term survival prognosis	Pathological risk factors	MVI
		Satellite nodule
		Differentiation
	Molecular Markers	CTCs/ctDNA
		NLR/PLR
		Specific genetic expression
	Immune markers	PD-L1 expression
		CD8^+^ T cells

Note: AFP, alpha-fetoprotein; DCP, des-gamma-carboxy prothrombin; NLR, neutrophil-to-lymphocyte ratio; PLR, platelet-to-lymphocyte ratio; ALBI, albumin–bilirubin; ICG R15, indocyanine green retention rate at 15 min; MVI, microvascular invasion; CTC, circulating tumor cell; ctDNA, circulating-tumor DNA.
